# Two Vaccines for *Staphylococcus aureus* Induce a B-Cell-Mediated Immune Response

**DOI:** 10.1128/mSphere.00217-18

**Published:** 2018-08-22

**Authors:** Christopher D. Dupont, Ingrid L. Scully, Ross M. Zimnisky, Brinda Monian, Christina P. Rossitto, Ellen B. O'Connell, Kathrin U. Jansen, Annaliesa S. Anderson, J. Christopher Love

**Affiliations:** a Koch Institute for Integrative Cancer Research, Department of Chemical Engineering, Massachusetts Institute of Technology, Cambridge, Massachusetts, USA; b Pfizer Vaccine Research and Early Development, Pearl River, New York, USA; c The Ragon Institute of MGH, MIT and Harvard, Cambridge, Massachusetts, USA; University of Nebraska Medical Center

**Keywords:** immunization, memory B cells, microengraving

## Abstract

Staphylococcus aureus causes severe disease in humans for which no licensed vaccine exists. A novel S. aureus vaccine (SA4Ag) is in development, targeting the capsular polysaccharides (CPs) and two virulence-associated surface proteins. Vaccine-elicited antibody responses to CPs are efficacious against serious infection by other encapsulated bacteria. Studies of natural S. aureus infection have also shown a role for T_H_17 and/or T_H_1 responses in protection. Single-antigen vaccines, including CPs, have not been effective against S. aureus; a multiantigen vaccine approach is likely required. However, the impact of addition of protein antigens on the immune response to CPs has not been studied. Here, the immune response induced by a bivalent CP conjugate vaccine (to model the established mechanism of action of vaccine-induced protection against Gram-positive pathogens) was compared to the response induced by SA4Ag, which contains both CP conjugates and protein antigens, in cynomolgus macaques. Microengraving, flow cytometry, opsonophagocytic assays, and Luminex technology were used to analyze the B-cell, T-cell, functional antibody, and innate immune responses. Both the bivalent CP vaccine and SA4Ag induced cytokine production from naive cells and antigen-specific memory B-cell and functional antibody responses. Increases in levels of circulating, activated T cells were not apparent following vaccination, nor was a T_H_17 or T_H_1 response evident. However, our data are consistent with a vaccine-induced recruitment of T follicular helper (T_F_H) cells to lymph nodes. Collectively, these data suggest that the response to SA4Ag is primarily mediated by B cells and antibodies that abrogate important S. aureus virulence mechanisms.

**IMPORTANCE** Staphylococcus aureus causes severe disease in humans for which no licensed vaccine exists. A novel vaccine is in development that targets multiple elements of the bacteria since single-component vaccines have not shown efficacy to date. How these multiple components alter the immune response raised by the vaccine is not well studied. We found that the addition of two protein components did not alter substantially the antibody responses raised with respect to function or mobilization of B cells. There was also not a substantial change in the activity of T cells, another part of the adaptive response. This study showed that protection by this vaccine may be mediated primarily by antibody protection.

## Abstract (precis)

Staphylococcus aureus causes severe disease in humans for which no licensed vaccine exists. A novel vaccine is in development that targets multiple elements of the bacteria since single-component vaccines have not shown efficacy to date. How these multiple components alter the immune response raised by the vaccine is not well studied. We found that the addition of two protein components did not alter substantially the antibody responses raised with respect to function or mobilization of B cells. There was also not a substantial change in the activity of T cells, another part of the adaptive response. This study showed that protection by this vaccine may be mediated primarily by antibody protection.

## INTRODUCTION

The Gram-positive bacterium Staphylococcus aureus is a pathogen of major importance to public health. While S. aureus commonly asymptomatically colonizes the skin and nose of healthy humans, severe disease can result from infection of the blood, bone, skin, and lungs, as well as sites of catheters and prosthetic material (1). The threat posed by S. aureus is further exacerbated by the development of strains resistant to methicillin (methicillin-resistant S. aureus [MRSA] strains) and other antibiotics that are estimated to have caused more than 75,000 infections and over 9,600 deaths in the United States in 2012 (2). Since there are currently no licensed vaccines against S. aureus infection, novel therapeutics to treat or prevent this disease are urgently needed.

To develop a vaccine against S. aureus, efforts have focused on identifying the mechanisms by which hosts resist this infection. Since S. aureus largely persists extracellularly, it is not surprising that *in vitro* studies, animal models, and the susceptibilities of patients with specific immunodeficiencies have collectively suggested that neutrophil-based killing is critical for controlling S. aureus infection. T_H_17 cells promote the recruitment and activation of neutrophils, which phagocytose and kill the bacteria; T_H_17 responses have been implicated in protection against cutaneous S. aureus infections (3–10). This phagocytic killing can result from direct recognition of the bacteria through innate receptors or antibody (Ab)-mediated phagocytosis (11–15). The role of antibodies in immunity to S. aureus also implies a role for T follicular helper (T_F_H) cells; this subset of T cells is essential for germinal center reactions and antibody affinity maturation and therefore controls the maturation of B cells and antibody production by plasma cells (16). Additionally, the idea of a potential role for T_H_1 immune responses is supported by the ability of S. aureus to persist intracellularly and by the observation that gamma interferon (IFN-γ) can activate neutrophil killing mechanisms (5, 17–20). Together, those studies demonstrated the role for adaptive immune responses in resistance to S. aureus and identified specific components of the immune system that may be useful to target with vaccination.

The identification of specific molecular antigens of S. aureus that the immune system can recognize has been another challenge for vaccine development (21–23). Like many bacterial pathogens, S. aureus can evade detection by innate immune receptors by encapsulating itself with polysaccharides (15, 24). Capsular polysaccharides (CPs) have been well established as effective targets for vaccines against encapsulated bacteria, such as Streptococcus pneumoniae, Haemophilus influenzae, and Neisseria meningitidis. As many as eight capsular serotypes have been identified in S. aureus, but the majority of disease-causing isolates express either capsular polysaccharide 5 (CP5) or CP8, making these attractive vaccine targets (25). Expression of capsular polysaccharides, however, can be dynamic during infection, and targeting additional protein antigens may be necessary for adequate protection (26). Two S. aureus surface proteins that have been identified as major virulence factors may serve as additional vaccine targets: manganese transport protein C (MntC) and clumping factor A (ClfA). MntC is involved in the scavenging of manganese ions, which are important both as nutrients for S. aureus and as cofactors for superoxide dismutase, which enables S. aureus survival of the neutrophil respiratory burst. ClfA promotes binding of S. aureus to platelets and fibrogen, which is necessary for disease pathogenesis in several models of infection with this pathogen (27–33). Preclinical studies have demonstrated the ability of these antigens to induce protection in animal models (32, 34–36). ClfA and MntC may therefore serve as valuable antigens to combine with capsular polysaccharide conjugates in a vaccine against S. aureus.

The identification of these antigens has led to the development of several candidate vaccines at various stages of development (21–23). Use of a bivalent vaccine containing the CP5 and CP8 polysaccharides conjugated to recombinant Pseudomonas aeruginosa exoprotein A showed reduced bacteremia in initial phase III clinical trials (37). However, a significant protective effect was not observed in a subsequent trial (38), which may have been due to the population chosen, suboptimal conjugate production, or differences in conjugate manufacture between trials (38). On the basis of the partial success of this trial and strong preclinical evidence for the use of capsular polysaccharide conjugates as vaccine candidates, a novel tetravalent vaccine (SA4Ag) has been designed that includes CP5 and CP8, each conjugated to cross-reacting material 197 (CRM_197_), in addition to the MntC and ClfA antigens. The effect that the addition of these protein antigens may have on the anti-CP immune response has not been studied in detail and is a topic of this publication.

To determine how the response to SA4Ag compares to the immune response induced by vaccination against CP conjugates alone, groups of cynomolgus macaques were vaccinated with either the bivalent CP vaccine (CP5-CRM_197_ or CP8-CRM_197_) or the SA4Ag vaccine and the induced immune responses were characterized. Functional antibody responses, quantities of memory B and T cells, and levels of cytokine production from preimmunization and postimmunization T cells and bulk peripheral blood mononuclear cells (PBMCs) were measured. Our results demonstrate that both the capsular polysaccharide antigens and the protein antigens are immunogenic and that both vaccines induced memory B-cell and functional antibody responses. In contrast, neither of the vaccines induced increases in the levels of circulating activated T cells (either T_H_1 or T_H_17), though both vaccines may have promoted recruitment of T follicular helper cells to lymph nodes. Collectively, these data suggest that the response to these vaccines could be driven by B cells and antibodies and that the inclusion of the surface protein antigens does not negatively affect the anti-CP immune response and does not induce a strong effector T cell response.

## RESULTS

### Analysis of innate immune responses induced by the CP5-CRM_197_ and CP8-CRM_197_ or MntC and ClfA antigens.

**FIG 1:**
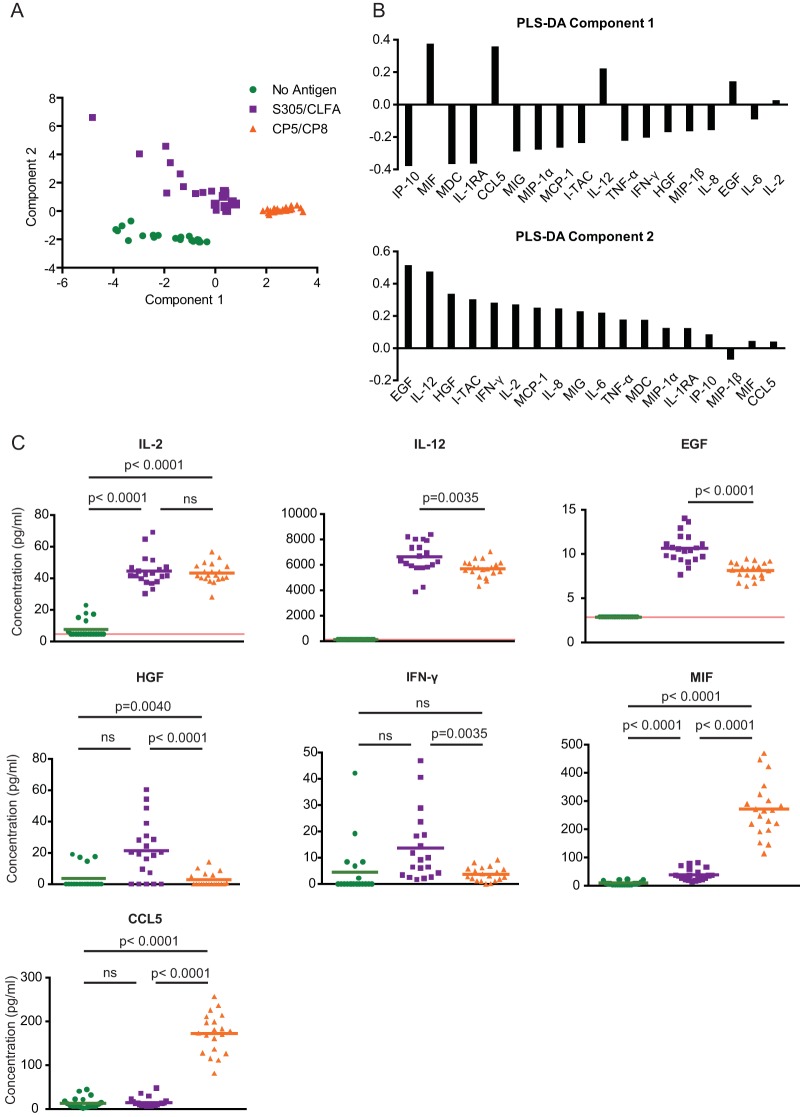
Analysis of innate antigen-induced cytokine responses. Cryopreserved PBMCs were cultured with no antigen, CP5 and CP8, or ClFA and MntC antigen. After incubation for 48 h, the concentrations of 29 cytokines in the supernatants were measured by Luminex analysis. Concentrations of all 18 cytokines with detectable signal were inputted into a partial least-squares discriminant analysis (PLS-DA) algorithm in order to identify key cytokines corresponding to each antigen stimulation condition. This algorithm outputted the data in a principal-component space, where each component represents an axis of maximal variance in the data and separation between stimulation conditions. (A) Each data point, plotted in the PLS-DA principal-component space, represents all 18 cytokine concentrations from an individual naive macaque under a specific antigen stimulation condition. The macaques maintained within each antigen stimulation condition clustered together, suggesting a consistent set of cytokines induced across all macaque PBMCs within a specific stimulation condition. (B) The loadings for each principal component indicate which cytokines corresponded to each antigen stimulation condition. For example, based on the loadings of principal component 1, the CP5 and CP8 antigens stimulated increased expression of MIF, CCL5, and IL-12. (C) Observed concentrations of individual cytokines are shown. Each data point represents an individual naive macaque under a specific antigen stimulation condition. The red lines indicate the limit of detection (LOD) for each cytokine; measurements at or below the LOD were excluded from statistical analysis. *P* values were calculated using a Mann-Whitney *U* test. ns, not significant.

Adaptive immune responses that confer protection following vaccination are ultimately regulated by the initial cytokines that are released when specific ligands are sensed by innate pattern recognition receptors (39). We sought to determine whether or not the innate cytokine responses induced by the MntC and ClfA antigens were distinct from those induced by the CP5-CRM_197_ and CP8-CRM_197_ antigens. PBMCs from unvaccinated macaques were cultured in the absence of antigen or in the presence of ClfA and MntC or of CP5-CRM_197_ and CP8-CRM_197_ antigens. The concentrations of 29 cytokines were measured in the supernatants after incubation. Partial least-squares discrimination analysis (PLS-DA) was applied to determine if the pairs of antigens elicited distinct cytokine responses and what cytokines distinguish these responses from one another. This analysis revealed that the macaque cells from each antigen stimulation condition clustered into distinct groups, distinguishable by the cytokines that they produced (Fig. 1A). Stimulation with MntC and ClfA or with both CP5-CRM_197_ and CP8-CRM_197_ induced secretion of the cytokines IL-2, IL-12, epidermal growth factor (EGF), and migration inhibition factor (MIF) (Fig. 1B and C). Stimulation with CP5-CRM_197_ and CP8-CRM_197_ also induced secretion of CCL5. In contrast, stimulation with MntC and ClfA did not induce secretion of CCL5. The two antigen pairs differed in the amounts of MIF and EGF that they induced. The ClfA and MntC antigen pairs also induced greater IFN-γ production than the CP5-CRM_197_ and CP8-CRM_197_ antigen pairs, although, correcting for the multiple comparisons being made, this trend was not statistically significant. Collectively, these data demonstrate that both antigen pairs (antigens CP5 and CP8 and antigens ClfA and MntC) are immunogenic and capable of eliciting cytokine responses but that the cytokine responses that they elicit are distinct from one another.

### Analysis of memory B-cell responses induced by bivalent vaccine treatment or SA4Ag vaccination.

**FIG 2:**
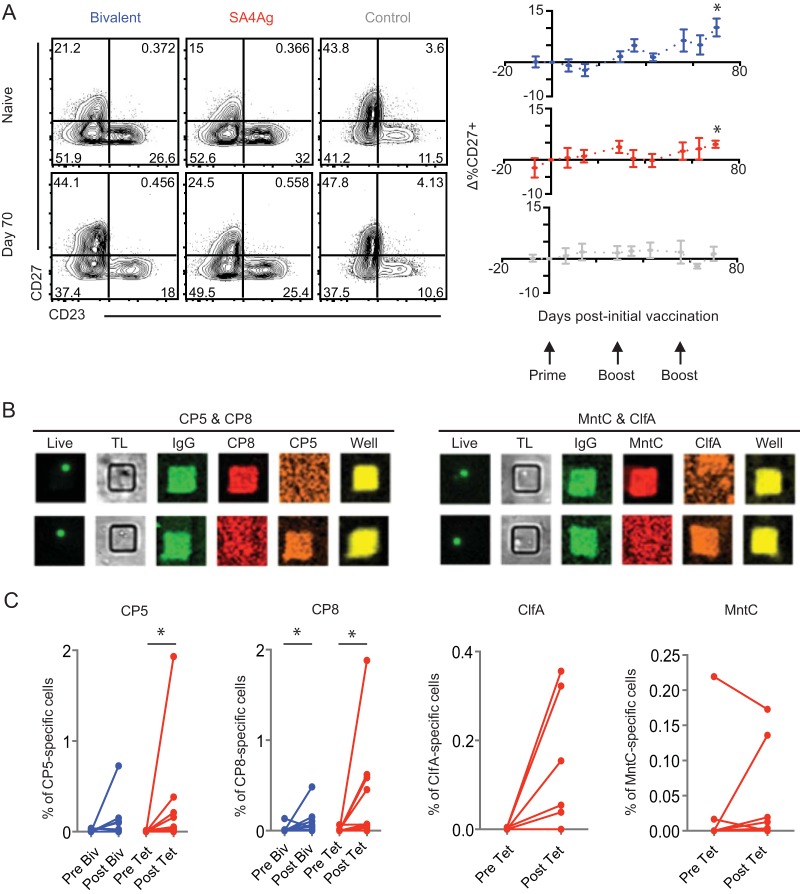
Memory B-cell responses to bivalent and SA4Ag vaccination. (A and B) Memory B-cell responses measured by flow cytometry. Cryopreserved PBMCs from vaccinated and unvaccinated macaques were thawed and stained with antibodies to identify activated B cells. Representative flow cytometry plots show the CD27 and CD23 expression of B cells (A). Flow plots are gated on singlet, live, CD20^+^ CD3^−^ CD56^−^ CD16^−^ cells. Kinetics of the memory B-cell response in each vaccination group are shown. Changes in CD27 expression were calculated by subtracting the percentage of B cells that were positive for CD27 at day 0 from the percentage of B cells that were positive for CD27 at each time point. Gray arrows represent the time points at which macaques were vaccinated. When samples from day 0 for a given macaque were unavailable, other time points were normalized to day −7 and the value representing the change in CD27 at day −7 (which was by definition 0) was excluded. All macaques were analyzed at each time point except when insufficient sample was available. A paired *t* test was used to assess significance of CD27^+^ B cell frequencies at each time point compared to day 0. A single asterisk (*) refers to an adjusted *P* value of <0.05 (Bonferroni correction). The *n* values for each group at each time point are as follows: bivalent, *n* = 9, 10, 4, 9, 9, 10, 8, 9, 10, and 9; SA4Ag, *n* = 7, 10, 8, 9, 9, 10, 5, 9, 7, and 7; control, *n* = 9, 6, 8, 8, 10, 9, 9, 8, 5, and 7. Means ± standard errors of the means (SEM) are shown. (B) Sample images showing microengraving signals. (C) Frequencies of antigen-specific B cells prevaccination and at day 70 postvaccination, determined by microengraving; “Biv” refers to bivalent vaccine, and “Tet” refers to tetravalent or SA4Ag vaccine. A Wilcoxon rank sum test was used to assess the significance of the results of comparisons between the prevaccination and postvaccination frequencies. A single asterisk (*) indicates significance at alpha = 0.05. The *n* values for each group for each antigen are as follows: bivalent, *n* = 9 and 10 (CP5 and CP8); SA4Ag, *n* = 9, 10, 5, and 5 (CP5, CP8, Clfa, and MntC). Insufficient numbers were present to perform the test in the cases of Clfa and MntC.

Memory B cells represent one component of the adaptive immune system that can confer vaccine-induced protection, and the vast majority of vaccines currently in use are thought to elicit B-cell responses (40). Therefore, we next compared the frequencies of memory B cells, and the relative binding affinities of the antibodies secreted by them, following bivalent vaccine treatment or SA4Ag vaccination. The kinetics of the memory B-cell response throughout the vaccination regimen was examined by flow cytometry, measuring expression of receptor CD27, which is upregulated on memory B cells, and receptor CD23, which is expressed on naive B cells (41). Analysis of these markers revealed three populations of B cells (CD23^+^ CD27^−^, CD23^−^ CD27^−^, and CD23^−^ CD27^+^). Of these, only the level of the CD23^−^ CD27^+^ population was found to have increased at day 70 following vaccination (Fig. 2A; see also Fig. S1 in the supplemental material). Vaccination with either the SA4Ag vaccine or the bivalent vaccine induced increases in the levels of memory B cells, which peaked at the end of the vaccination regimen. These data suggest that inoculation with either the SA4Ag vaccine or the bivalent vaccine elicits memory B-cell responses.

10.1128/mSphere.00217-18.1 FIG S1  Vaccine-induced changes in subsets of the B-cell population. B cells were obtained and treated as described for Fig. 1. Representative flow cytometry plots (gated on B cells) are shown, as are the changes in the percentage of each subset of B cells determined by CD23 and CD27 expression. Percentages were calculated as described for Fig. 1. Download FIG S1, PDF file, 0.2 MB. Copyright © 2018 Dupont et al. 2018 Dupont et al. This content is distributed under the terms of the Creative Commons Attribution 4.0 International license.

Another important aspect of the B-cell response to assess is antigen specificity. To compare the antigen-specific memory B-cell responses elicited by each vaccine, PBMCs collected before vaccination and at day 70 postvaccination were stimulated to induce antibody secretion. The levels of secretion of antibodies and their specificities were then measured using microengraving. After stimulation, cells were deposited into an array of subnanoliter wells (50 µm by 50 µm by 100 µm); the antibodies secreted by individual B cells within each well were then captured on a glass slide and probed with fluorescently labeled antigens and detection antibodies (42). Using this technique, B cells that secreted antibodies could be clearly identified, along with the specificity of the antibodies for each vaccine component (Fig. 2B). Using this approach, we observed that both bivalent CP and SA4Ag induced increases in the frequencies of CP5- and CP8-specific B cells and that SA4Ag induced increases in the frequencies of ClFA- and MntC-specific B cells in the majority of macaques examined. Comparing the frequencies of CP5- and CP8-specific memory B cells from prevaccination and 70 days postvaccination revealed that the bivalent CP and SA4Ag vaccines elicited similar increases in the frequencies of antigen-specific memory B cells (Fig. 2C).

### Analysis of T-cell responses induced by bivalent vaccine treatment or SA4Ag vaccination.

**FIG 3:**
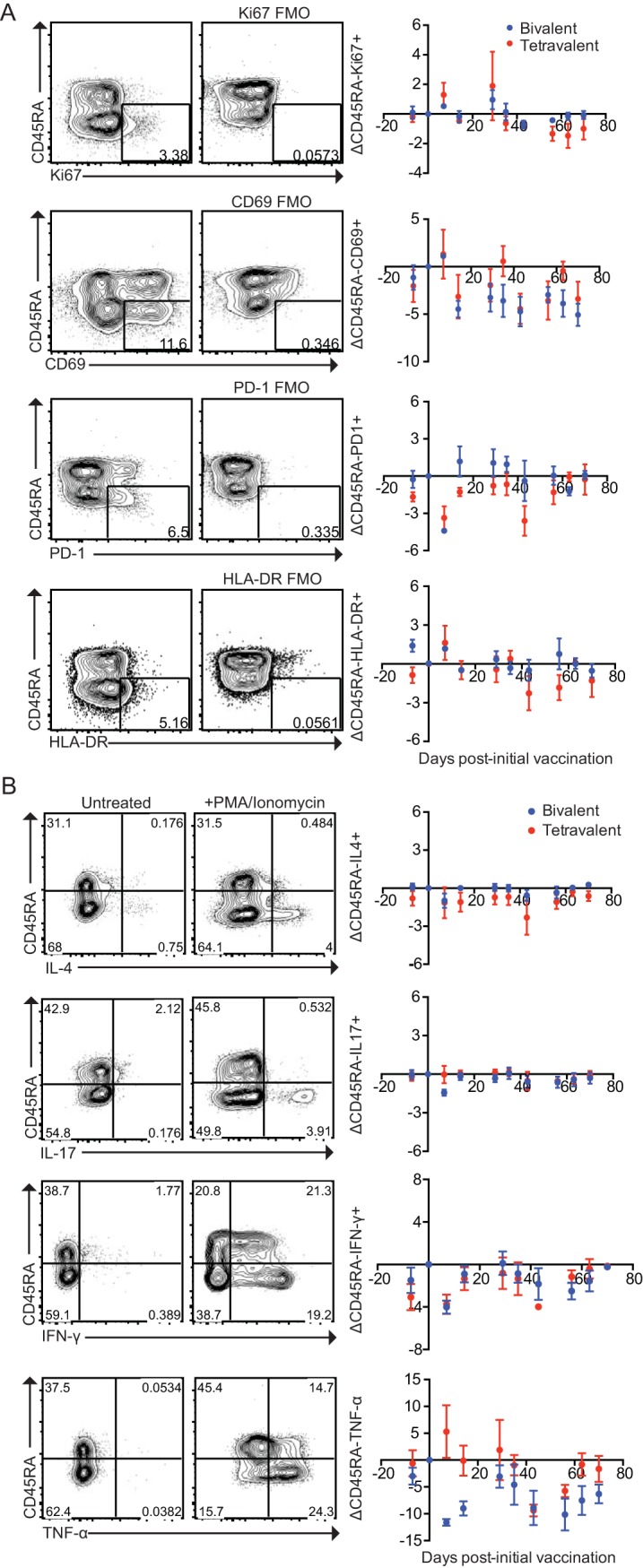
Examination of T-cell activation status and cytokine production before and after vaccination. (A) Expression of activation markers on T cells at various time points following vaccination. Expression of CD45RA, Ki-67, CD69, PD-1, and HLA-DR on the surface of T cells was examined. Representative flow cytometry plots are shown. Flow plots are gated on singlet, live, CD3^+^ CD4^+^ T cells. Changes in expression of each activation marker represent the difference between the percentage of T cells positive at each time point and the percentage at day 0. When samples from day 0 for a given macaque were unavailable, other time points were normalized to day −7 and the value representing the change in CD27 at day −7 (which was by definition 0) was excluded. The data shown represent an analysis of half of the population of macaques used for this study. PBMCs from the other half of the population of macaques were stained with a slightly different panel, and these data are shown in Fig. S2. Macaques were excluded only when insufficient sample was available. *N* values for each time point are as follows: bivalent, *n* = 5, 5, 1, 5, 5, 5, 4, 5, 5, and 5; SA4Ag, *n* = 4, 4, 3, 4, 4, 5, 2, 3, 2, and 3. Means ± SEM are shown. FMO, fluorescence minus one. (B) Production of cytokines from bivalent and SA4Ag-vaccinated macaques at various time points, measured by intracellular cytokine staining. PBMCs were stimulated for 4 h with PMA and ionomycin at a concentration of 1 µg/ml and BD GolgiStop according to the manufacturer’s instructions. Representative flow cytometry plots are shown. Plots are gated on singlet, live, CD3^+^ CD4^+^ T cells. Changes in expression of each activation marker represent the difference between the percentage of T cells positive at each time point and the percentage at day 0. When samples from day 0 for a given macaque were unavailable, other time points were normalized to day −7 and the value representing the change in CD27 at day −7 (which was by definition 0) was excluded. The data shown represent an analysis of half of the population of macaques used for this study. PBMCs from the other half of the population of macaques were stimulated in an earlier experiment with a lower concentration of PMA and ionomycin (0.1 µg/ml), and these data are shown in Fig. S2. Macaques were excluded only when insufficient sample was available. *N* values for each time point are as follows: bivalent, *n* = 5, 5, 2, 5, 5, 5, 4, 3, 5, and 5; SA4Ag; *n* = 5, 5, 3, 3, 4, 5, 2, 4, 2, and 3. Means ± SEM are shown.

In addition to memory B cells, T cells represent another compartment of the adaptive immune system that may be involved in protection following vaccination. T-cell-mediated immunity has been implicated in resistance to S. aureus (3–5) and has also been negatively linked to vaccine safety (43). We used several approaches to measure the vaccine-induced T cell responses. First, T cells taken from macaques were analyzed *ex vivo* by flow cytometry for vaccine-induced changes in the expression of activation-associated molecules. Specifically, T cells were examined for expression of surface molecules CD69, PD-1, and HLA-DR (which are upregulated on recently activated CD4^+^ T cells) (44, 45); surface molecule CD45RA (which is downregulated on antigen-experienced CD4^+^ T cells) (46); and intracellular protein Ki-67 (which is associated with cellular proliferation) (47). Flow cytometric analysis revealed no changes in the expression of any of these molecules (Fig. 3A; see also Fig. S2A) following bivalent CP or SA4Ag vaccination, arguing against a prominent role for circulating T-cell populations in vaccine-induced immunity.

10.1128/mSphere.00217-18.2 FIG S2  Analysis of T cell phenotype and function before and after vaccination. (A) Expression of activation markers on T cells at various time points following vaccination. Expression of CD45RA, Ki-67, and PD-1 on the surface of T cells was examined. Representative flow cytometry plots are shown. Flow plots are gated on singlet, live, CD3^+^ CD4^+^ T cells. Changes in expression of each activation marker represent the difference between the percentage of T cells positive at each time point and the percentage at day 0. When samples from day 0 for a given macaque were unavailable, other time points were normalized to day −7 and the value representing the change in CD27 at day −7 (which was by definition 0) was excluded. The data shown represent an analysis of half of the population of macaques used for this study. PBMCs from the other half of the population of the macaques were stained with a slightly different panel, and these data are shown in Fig. 3. Macaques were excluded only when insufficient sample was available. *N* values for each time point are as follows: bivalent, *n* = 5, 5, 5, 5, 4, 5, 4, 5, 5, and 4; SA4Ag, *n* = 3, 5, 4, 5, 5, 5, 4, 5, 5, and 5; control, *n* = 4, 5, 3, 4, 5, 5, 5, 3, 3, and 4. Means ± SEM are shown. (B) Production of cytokines from bivalent and SA4Ag-vaccinated macaques at various time points, measured by intracellular cytokine staining. PBMCs were stimulated for 4 h with PMA and ionomycin at a concentration of 0.1 µg/ml and BD GolgiStop according to the manufacturer’s instructions. Representative flow cytometry plots are shown. Plots are gated on singlet, live, CD3^+^ CD4^+^ T cells. Changes in expression of each activation marker represent the difference between the percentage of T cells positive at each time point and the percentage at day 0. When samples from day 0 for a given macaque were unavailable, other time points were normalized to day −7 and the value representing the change in CD27 at day −7 (which was by definition 0) was excluded. The data shown represent an analysis of half of the population of macaques used for this study. PBMCs from the other half of the population of macaques were stimulated in another experiment with a higher concentration of PMA and ionomycin (1 µg/ml), and these data are shown in Fig. 3. Macaques were excluded only when insufficient sample was available. *N* values for each time point are as follows: bivalent, *n* = 5, 5, 4, 5, 4, 5, 4, 5, 5, and 4; SA4Ag, *n* = 3, 5, 4, 5, 4, 5, 4, 5, 5, and 4; control, 4, 5, 3, 4, 5, 5, 5, 4, 3, and 4. Means ± SEM are shown. Download FIG S2, PDF file, 0.3 MB. Copyright © 2018 Dupont et al. 2018 Dupont et al. This content is distributed under the terms of the Creative Commons Attribution 4.0 International license.

**FIG 4:**
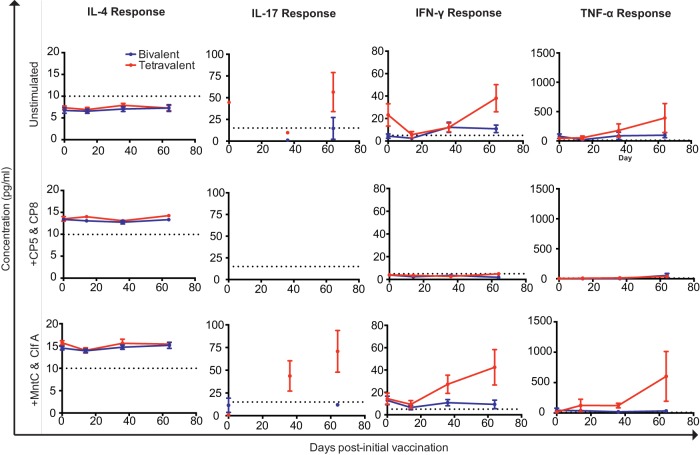
Cytokine production resulting from antigen stimulation. Cryopreserved PBMCs from each vaccine group, at each time point, were thawed, rested overnight, and cultured at a concentration of 500,000 cells/well in 90 µl of AIM-V media per well. Cells were stimulated with 8 µg/ml of MntC and ClfA or CP5 and CP8 antigens. Supernatants were harvested and examined for cytokine content after 48 h of stimulation. Limits of detection for each cytokine (as reported by the manufacturer) are depicted with the dashed line. Points falling below the standard curve were excluded. Other samples were excluded only when insufficient sample was present. *N* values for each time point are as follows: bivalent, no antigen, *n* = 10, 10, 10, and 9; SA4Ag, no antigen, *n* = 10, 10, 10, and 10; bivalent, CP5 and CP8 stimulation, *n* = 10, 10, 10, and 10; SA4Ag, CP5 and CP8 stimulation, *n* = 10, 10, 10, and 10; bivalent, MntC and ClfA stimulation, *n* = 10, 9, 10, and 4; SA4Ag, MntC and ClfA stimulation, *n* = 10, 9, 9, and 7.

To further assess vaccine-induced immune responses, intracellular cytokine staining (ICS) was performed to determine whether bivalent CP or SA4Ag vaccination increased cytokine production. Expression levels of tumor necrosis alpha (TNF-α), IFN-γ, and IL-17 were measured; all of these cytokines have been implicated in immunity to S. aureus (5, 6, 18, 48, 49). Expression of IL-4 was also measured, since this cytokine can promote B-cell responses (16). After stimulating cryopreserved PBMCs from macaques with phorbol myristate acetate (PMA) and ionomycin to induce activation, no vaccine-induced increases in cytokine production were observed (Fig. 3B; see also Fig. S2B). In a further effort to detect antigen-specific cytokine responses, cryopreserved PBMCs from multiple pre- and postvaccination time points were cultured with the vaccine components, and the concentrations of an array of 29 cytokines (including IL-4, IL-17, TNF-α, and IFN-γ) were then measured. While treatment of the PBMCs with antigens induced increases in the secretion of a number of cytokines regardless of the time point postvaccination, no evidence of vaccine-induced increases in cytokine secretion was apparent using this method (Fig. 4; see also Fig. S3). Collectively, these results suggest that neither the bivalent CP nor SA4Ag induced significant activation of circulating T cells.

10.1128/mSphere.00217-18.3 FIG S3  Antigen-induced cytokine production before and after vaccination. To further interrogate the changes in cytokine production from PBMCs induced by vaccination, PLS-DA was applied to analyze the production of the 29 cytokines examined, generating component scores for each macaque at each time point. No changes in the vaccine-induced cytokine signatures (represented by component 1 and component 2) were apparent at any time point. Cytokine production from PBMCs was measured as described for Fig. 4. Download FIG S3, PDF file, 0.04 MB. Copyright © 2018 Dupont et al. 2018 Dupont et al. This content is distributed under the terms of the Creative Commons Attribution 4.0 International license.

**FIG 5:**
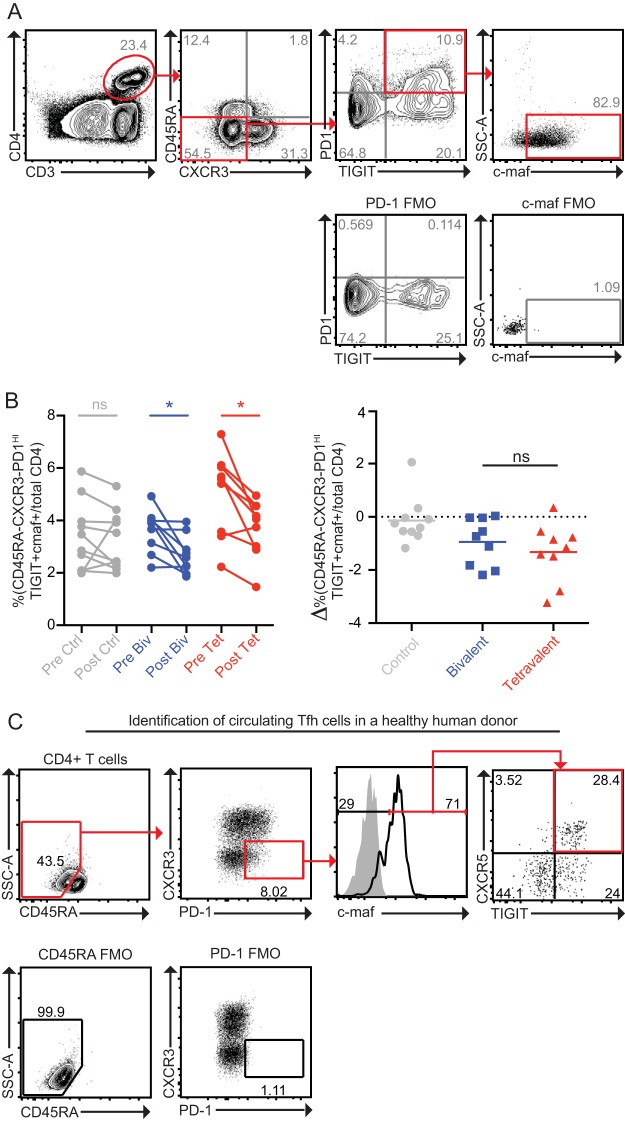
Vaccination induces a decrease in CD4^+^ CD45RA^−^ CXCR3^−^ PD-1^Hi^ TIGIT^+^ c-maf^+^ T cells. (A and B) Cryopreserved T cells from days 0 and 70 post-initial vaccination were thawed and rested overnight. Expression of markers for T follicular helper cells was measured on each. A representative gating strategy to identify CD4^+^ CD45RA^−^ CXCR3^−^ PD-1^HI^ TIGIT^+^ c-maf^+^ T cells is shown (A), and the frequencies of these cells as a percentage of the total CD4^+^ T-cell population were quantified (B). Ctrl, control or vaccine buffer; Biv, bivalent vaccine; Tet, tetravalent vaccine. (C) A representative gating strategy to identify circulating TFH cells in a human subject is shown.

Recent studies have demonstrated that T follicular helper cells (T_F_H), a cellular population specialized in promoting B-cell responses, can be detected in circulation and that they bear a CD4^+^ CD45RA^−^ CXCR3^−^ CXCR5^+^ PD-1^HI^ TIGIT^HI^ c-maf^HI^ phenotype (50). Identification of these cells in macaques is complicated by the low levels of detectable CXCR5 expressed on macaque T cells (51). However, a population of CD4^+^ CD45RA^−^ CXCR3^−^ PD-1^HI^ TIGIT^+^ c-maf^+^ cells could be detected in the macaque’s circulation. Vaccination with either the SA4Ag or bivalent CP vaccine (but not control buffer) induced significant decreases in this population (Fig. 5A and B). No significant differences were apparent when the frequencies of these cells were compared between the bivalent and SA4Ag vaccine groups. Importantly, when this gating strategy was applied to human PBMCs, a majority of cells within the CD4^+^ CD45RA^−^ CXCR3^−^ PD-1^HI^ TIGIT^+^ c-maf^+^ population were found to be CXCR5^+^ (i.e., were circulating T_F_H cells) (Fig. 5C). These results are therefore consistent with a model in which vaccination induces a decrease in the circulating T_F_H cell population, possibly due to the recruitment of circulating T_F_H cells to lymph nodes.

### Effects of vaccination on humoral immune responses.

To measure the functional properties of the antibody responses induced by vaccination with the bivalent and SA4Ag macaques, sera from each macaque were collected at multiple time points and analyzed in two ways: the ability of the sera to opsonize S. aureus for phagocytosis by neutrophils *in vitro* was determined using an opsonophagocytic assay (OPA), and the ability of antibodies from the sera to prevent binding of fluorescently labeled monoclonal antibodies (Abs) to the antigenic vaccine components was determined using a competitive Luminex immunoassay (cLIA) (52).

**FIG 6:**
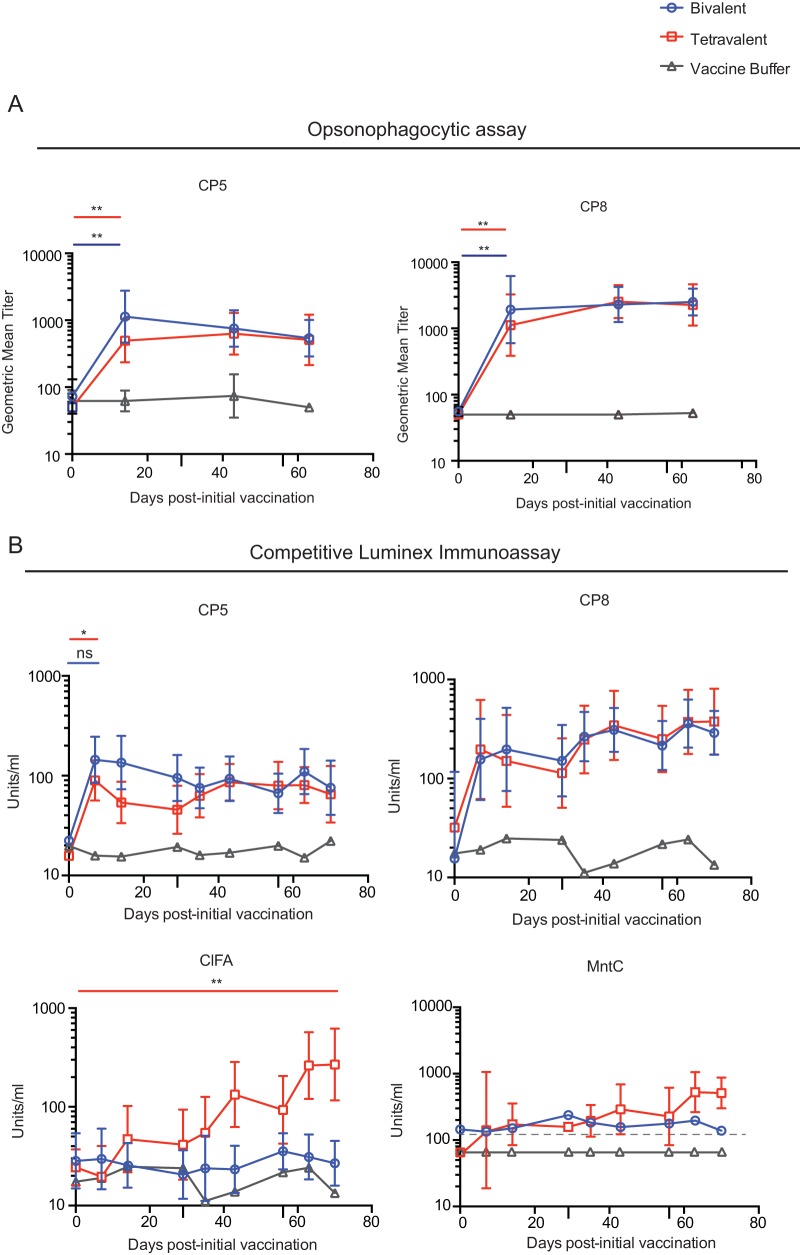
Functional analysis of antibodies in the sera induced by the bivalent vaccine-treated or SA4Ag vaccines. Sera were harvested from bivalent vaccine-treated, SA4Ag-vaccinated, or control buffer-vaccinated macaques at the indicated time points. (A) Analysis of opsonophagocytic activity. Sera were diluted and incubated with CP5-expressing or CP8-expressing isolates of S. aureus and human polymorphonuclear leukocytes overnight. The amount of bacteria present in each culture was quantified the following morning. Titers at which 50% of the bacteria were killed are reported. Titers represent the reciprocal of the serum dilution. Geometric mean titers ± 95% confidence intervals are shown. Values which fell below the limit of detection (where no detectable opsonization occurred) have been entered as 50. The geometric mean titer at which 50% of the bacteria were killed following vaccination (at day 14) is compared to the geometric mean titer at which 50% of the bacteria were killed prior to vaccination at day 0 within each group using a Wilcoxon signed-rank test. The geometric mean titer at which 50% of the bacteria were killed at day 14 in each group was compared to the geometric mean titer at which 50% of the bacteria at day 14 were killed in each other group using a Mann-Whitney *U* test (values not shown). (B) Antigen-binding ability of antibodies in sera measured by competitive Luminex immunoassay. Concentrations of sera (expressed in units per milliliter) at which 50% inhibition of the monoclonal antibody to its cognate vaccine component antigen occurred are displayed. Concentrations are displayed as geometric means ± 95% confidence intervals. To improve image clarity, the confidence intervals for the control buffer-vaccinated group were excluded. For visualization purposes, any conditions where all donors had values below the limit of detection (LOD) are plotted at half of the lower limit of quantitation (LLOQ) for visualization purposes, and the LLOQ is shown as a dashed line. When a sufficient number of macaques in each group had sera which detectably inhibited binding activity at day 0, the concentrations at which inhibition of binding occurred at day 0 and following vaccination were compared using a Wilcoxon matched-pair rank test. When a sufficient number of macaques in the control buffer-vaccinated group had sera which detectably inhibited binding activity following vaccination, the concentration at which binding was inhibited in this group was compared to the concentration at which it was inhibited in the bivalent vaccine-treated group or the SA4Ag-vaccinated group using a Mann-Whitney *U* test (values not shown).

The OPA revealed that vaccination with either the bivalent CP or SA4Ag induced a rapid increase in the ability to opsonize both CP5-expressing and CP8-expressing isolates of S. aureus. This outcome was apparent in comparisons of sera of macaques from day 0 to sera from day 14 post-initial vaccination and in comparisons of vaccinated macaques to nonvaccinated control groups (Fig. 6A; see also Fig. S4A). Both the percentage of macaques capable of opsonizing S. aureus and the titer at which opsonization occurred were significantly increased by vaccination with either the bivalent CP or SA4Ag. The increased ability to opsonize bacteria was sustained throughout the vaccination regimen in both bivalent CP-immunized and SA4Ag-immunized animals. No significant differences were apparent between the bivalent CP- and SA4Ag-vaccinated animals in their ability to opsonize S. aureus or in the titers at which opsonization occurred. Collectively, these results suggest that both the bivalent CP and SA4Ag vaccines induce humoral immune responses that are comparable with respect to their ability to opsonize S. aureus.

10.1128/mSphere.00217-18.4 FIG S4  Percentages of macaques showing opsonophagocytic activity or antigen-specific antibodies as measured by CLIA. (A) Percentages of macaques with sera capable of opsonizing S. aureus expressing CP5 (left) or CP8 (right) at any serum dilution. The percentages of macaques in each group whose sera displayed or did not display opsonophagocytic activity following vaccination (at day 14) were compared to the percentages of macaques whose sera displayed or did not display opsonophagocytic activity prior to vaccination (at day 0) using Fisher’s exact test. The percentages of macaques in each group whose sera displayed or did not display opsonophagocytic activity following vaccination (at day 14) were compared to the percentages of macaques whose sera displayed or did not display opsonophagocytic activity in each other group using Fisher’s exact test (values not shown). (B) Percentages of macaques whose sera could detectably inhibit the binding of a monoclonal antibody to its cognate vaccine component antigen are shown on the left. The percentages of macaques in each group which had detectable antigen-binding activity following vaccination were compared to the percentages which had detectable antigen binding activity prior to vaccination using Fisher’s exact test. The percentage of macaques that had detectable antigen-binding activity in the bivalent vaccine-treated or SA4Ag-vaccinated groups was compared to the percentage that responded in the control buffer-vaccinated group using Fisher’s exact test (values not shown). Download FIG S4, PDF file, 0.05 MB. Copyright © 2018 Dupont et al. 2018 Dupont et al. This content is distributed under the terms of the Creative Commons Attribution 4.0 International license.

Humoral immune responses to vaccination were also assessed using cLIA, which measures the ability of antibodies in the sera to inhibit the binding of fluorescently labeled monoclonal antibodies specific for the antigenic components of the vaccine (53). Using this approach, increases in the levels of CP5- and CP8-specific antibodies were apparent in both bivalent CP- and SA4Ag-immunized macaques following vaccination, as indicated by significant increases in the percentages of macaques whose sera were able to inhibit the binding of monoclonal antibodies to their cognate antigen and in the titers at which binding was inhibited (Fig. 6B; see also Fig. S4B). The CP5- and CP8-specific antibody responses induced by the SA4Ag and bivalent CP vaccines were sustained throughout the vaccination regimen. When the responses induced by the SA4Ag vaccine and bivalent CP vaccine were compared to one another using a 2-way analysis of variance (ANOVA), the anti-CP5 responses were found to be significantly different from one another. Comparisons between the SA4Ag- and bivalent CP-vaccinated macaques at each individual time point, however, revealed no significant differences (once corrections were made for performing multiple comparisons). No differences in the vaccine-induced anti-CP8 response were apparent between the bivalent CP- and SA4Ag-immunized macaques, as measured by the cLIA. The cLIA also revealed that antibody responses against ClfA and MntC were induced by vaccination with the SA4Ag vaccine but not by vaccination with the bivalent CP vaccine, as expected. The humoral responses against these protein antigens generally exhibited a kinetics different from that of the responses against the polysaccharide antigens and were slower to develop. Additionally, MntC antibody responses were difficult to detect by cLIA (although this may be a species-specific phenomenon, as cLIA-based responses to MntC are generally robust in humans [53]). Collectively, these results demonstrate that both the SA4Ag and bivalent CP vaccines induce functional humoral immune responses against all the antigenic components of each respective vaccine and show no evidence that the inclusion of the protein surface antigens negatively affects the anti-CP humoral response.

## DISCUSSION

Several previous efforts to develop vaccines for S. aureus have been unsuccessful (22). These approaches have targeted single antigens or targeted combinations of CPs (54). Given that expression of individual antigens is dynamic during infection, use of a multivalent vaccine targeting both CPs and surface antigens may be the most effective vaccination strategy (26). This strategy, however, raises the following question: how would inclusion of additional antigens affect the anti-CP immune response?

The studies performed here interrogated the effect that inclusion of additional surface antigens (ClfA and MntC) has on the immune responses against CPs. We observed no evidence that the anti-CP immune response was inhibited by the addition of the protein surface antigens. Indeed, the macaques exhibited similar frequencies of CP-specific memory B cells regardless of whether they were vaccinated with CP5 and CP8 alone or in combination with MntC and ClfA. Similarly, antibody responses (measured by cLIA or OPA) were not impaired by the addition of the ClfA and MntC antigens. Clinical data have confirmed that when MntC and ClfA are included in a multivalent vaccine, robust immune responses are observed (53, 55). Our data therefore suggest that the use of multivalent vaccines may be a promising strategy to prevent infection with S. aureus.

The results of this study also demonstrate that antigens ClfA and MntC and antigens CP5-CRM_197_ and CP8-CRM_197_ are antigen pairs capable of eliciting cytokine production from naive PBMCs and that both also induced antibody and memory B-cell responses following vaccination. These results are consistent with previous studies demonstrating the immunogenicity of CP5-CRM_197_, CP8-CRM_197_, MntC, and ClfA in healthy human subjects (53, 56). Interestingly, the antigen pairs induced distinctly different cytokine signatures in naive PBMCs, with CP5-CRM_197_ and CP8-CRM_197_ stimulation causing very high levels of MIF and CCL5 and with ClfA and MntC stimulation leading to elevated levels of EGF, hepatocyte growth factor (HGF), IFN-γ, and IL-12. These results suggest that the two antigen pairs have immune stimulatory effects that are distinct, which might play a subtle role in shaping the vaccine-induced immune response. Overall, the inherent immunogenicity of these antigens is important, as the SA4Ag vaccine currently in development contains no adjuvant.

It is presently unclear whether inducing T_H_17 or T_H_1 responses is a prudent strategy to prevent infection with extracellular pathogens. While local T_H_17 responses promote neutrophil migration to the site of infection, T_H_17 cells also can promote autoimmune responses (7). Likewise, while some researchers have described a protective role for T_H_1 responses in S. aureus infection following exposure to live S. aureus (20), a recent report has shown that induction of a T_H_1 response after vaccination with S. aureus may be deleterious (43). Our results found no evidence that vaccination with either the bivalent CP or SA4Ag vaccines induces T_H_1 or T_H_17 immune responses, suggesting that a similar result may also be observed in the clinic. As the majority of vaccines currently in use are thought to mediate protection primarily through antibody responses (40), it is reasonable to speculate that these memory T_H_1 or T_H_17 cell responses may not be necessary for protective immunity against S. aureus and could potentially be dangerous. Eliciting B-cell responses without T_H_1 or T_H_17 cells may therefore be ideal, as this strategy would mitigate concerns about the potential of vaccines to increase the risk for autoimmune disease and enhanced systemic inflammatory responses upon infection. Regardless, a greater understanding of the extent to which T_H_17 cells that are not self-reactive contribute to autoimmunity may help to inform future vaccine design.

Previous studies have measured circulating T_F_H cells and identified correlations between the frequencies of these cells and various phenotypes, such as the presence of broadly neutralizing antibodies in HIV patients (50, 57). Our studies identified a decrease in CD4^+^ CD45RA^−^ CXCR3^−^ PD-1^HI^ TIGIT^+^ c-maf^+^ levels induced by vaccination, and experiments using human PBMCs demonstrated that this population contains circulating T_F_H cells. Our results, however, identified no correlations between the decreases in this cell population and other parameters of the immune response, such as the increase in the frequency of memory B cells induced by vaccination or in the antibody responses, as measured by cLIA or OPA (data not shown). Regardless, our results implicate T_F_H involvement in the vaccine-induced immune response; this finding is consistent with current models in which T_F_H cells are thought to be necessary for class-switching and memory B cell development. Analyzing this population with greater resolution should be possible in human subjects, as CXCR5 is more easily detected on human T cells than on macaque T cells (51). Studies in human subjects may therefore reveal an important role for this population in the context of vaccine-induced immunity to S. aureus.

## MATERIALS AND METHODS

### Animals and immunization.

All animal protocols employed in this study met the established Pfizer Institutional Animal Care and Use Committee guidelines, and all animal work was conducted in an AALAC-accredited facility. Thirty cynomolgus macaques (Macaca fascicularis) were vaccinated intramuscularly with the bivalent vaccine (10 µg CP5-CRM_197_ and 10 µg CP8-CRM_197_), the SA4Ag vaccine (10 µg CP5-CRM_197_, 10 µg CP8-CRM_197_, 20 µg ClfA, and 20 µg MntC), or control vaccine buffer without adjuvant (*n* = 10) on days 0, 29, and 56 of the study. Blood was drawn −7, 0, 7, 14, 29, 35, 43, 56, 63, and 70 days post-initial vaccination. Peripheral blood mononuclear cells (PBMCs) were isolated from whole blood and stored at −200°C.

### Cell preparation.

Cells were thawed by briefly incubating them in a 37°C water bath and were added dropwise to room temperature AIM-V media (Thermo Fisher) containing 20 units/ml Benzonase (Novagen). Cells were washed extensively and rested overnight at 37°C in AIM-V media supplemented with 10% fetal bovine serum (FBS) (Gibco) for flow cytometry and Luminex experiments or were stimulated for 5 days in media (0.25 × 10^6^ cells/ml) supplemented with 10% FCS, 2.5 µg/ml R848 (InvivoGen), and 1,000 U/ml IL-2 (PeproTech) for microengraving experiments (58).

### Flow cytometry.

The following antibodies were used for flow cytometric analysis: brilliant UV (BUV) 737 anti-human IgG antibody (BD; clone G18-145), BUV395 mouse anti-human IgM antibody (BD; clone G20-127), polyclonal goat anti-human IgA antibody (Jackson; product number 109-005-149), phycoerythrin (PE) anti-macaque CD38 antibody (Nonhuman Primate Reagent Resource; clone OKT10), brilliant violet (BV) 605 anti-human CD20 antibody (BioLegend; clone 2H7), BV785 anti-human CD27 antibody (BioLegend; clone O323), Alexa Fluor (AF) 488 anti-human CD16 antibody (BioLegend; clone 3G8), AF488 anti-human CD56 antibody (BioLegend; clone MEM-188), AF488 anti-human CD3 antibody (BD; clone SP34), PE-Cy7 CD23 antibody (BD; clone M-L233), BV711 anti-human CD27 antibody (BioLegend; clone O323), BUV395 anti-human CD3 antibody (BD; clone 563563), allophycocyanin-H7 (APC-H7) anti-human CD4 antibody (BD; clone L200), fluorescein isothiocyanate (FITC) anti-human CD45RA antibody (Miltenyi; clone T6D11), BV785 anti-human PD-1 antibody (BioLegend; clone EH12.2H7), BV605 anti-human CXCR3 antibody (BD clone 1C6 and BioLegend clone GO25H7), PerCP-Cy5.5 anti-human CD69 antibody (BioLegend; clone fn50), BUV737 HLA-DR antibody (BioLegend; clone L243), APC anti-human TIGIT antibody (EBioscience; clone MBSA43), Percp-Cy5.5 anti-Ki-67 antibody (BD; clone B56), BUV395 anti-human IFN-γ antibody (BD; clone B27), PE anti-human IL-17 antibody (BD; clone SCPL1362), APC anti-human IL-4 antibody (BioLegend; clone 8D4-8), PE-Cy7 anti-human TNF-α antibody (BioLegend; clone MAb11), and PE anti-human/mouse c-maf antibody (EBioscience; clone sym0F1).

Human gamma globulin (Jackson) or fuman Fc block (BD) was used to inhibit binding to Fc receptors. The polyclonal anti-human IgA antibody was conjugated to PE-Cy7 using a conjugation kit (Abcam, Inc.; product number ab102903). A Zombie Violet fixable viability kit (BioLegend; product number 423114) was used to discriminate live and dead cells. Cells were stained for 25 min at 4°C for all extracellular surface stains. Staining was performed in Brilliant Stain buffer (BD). After staining, cells were washed with fluorescence-activated cell sorter (FACS) buffer (1× phosphate-buffered saline [PBS] supplemented with 0.2% bovine serum albumin [BSA; Sigma-Aldrich] and 1 mM EDTA [Promega]). Cells were fixed by incubation in 2% paraformaldehyde (PFA) (EMS) for 10 min. Cells were suspended in FACS buffer and analyzed using an LSR Fortessa flow cytometer. Data analysis was performed using FlowJo software (TreeStar) and Prism (GraphPad).

### Microengraving.

Microengraving protocols were performed following previously described methods (59). For microengraving analysis of macaque PBMCs, 25 µg/ml of polyclonal anti-human IgA plus IgG plus IgM (Jackson ImmunoResearch) and 25 µg/ml anti-rabbit IgG (Jackson ImmunoResearch) diluted in borate buffer were pipetted onto poly-lysine-coated slides, sealed with a coverslip, and incubated overnight in a humidity chamber at 4°C. Arrays comprising ~10^5^ wells (50 µm by 50 µm by 100 µm) were prepared as previously described (60). Following stimulation to induce antibody production, cells were enriched for antibody-secreting cells using negative magnetic selection (EasySep human B cell enrichment kit; StemCell Technologies) and were pipetted onto the wells at a density of ~1 cell per well, allowed to settle, sealed with the anti-IgG-coated slides, and incubated for 1 h at 37°C. Rabbit serum (Sigma-Aldrich) was added to the cells at a concentration of 1:8,000. Slides were subsequently probed with 5 µg/ml of AF488-labeled anti-human IgG antibody (BD; clone G18-145), AF700-labeled goat-anti rabbit antibody (Life Technologies, Inc.), and fluorescently labeled antigens (AF555-labeled CP5-HSA conjugate with AF647-labeled CP8-HSA conjugate or AF555-labeled MntC with AF647-labeled ClfA). All antigens were obtained from Pfizer and used at a concentration of 50 µg/ml. All antigens were conjugated to dyes using sodium bicarbonate before processing through fluorescent dye-removal columns (Pierce Biotechnology) was performed. Alexa Fluor fluorophores were obtained from Life Technologies, Inc.

Slides were washed and scanned using a GenePix 4400A microarray scanner (Molecular Devices). The images generated were analyzed using Crossword software (61) in addition to software developed in-house (T. M. Gierahn, unpublished data). Positive fluorescent events were identified based on the signal-to-noise ratio and average pixel intensity and were confirmed by visual inspection. Wells containing single cells were analyzed to determine the percentage of cells that secreted immunoglobulin. This frequency was multiplied by the total number of cells on each nanowell array (including wells that contained more than 1 cell) to determine the number of cells that secreted immunoglobulin. Numbers of antigen-specific spots were divided by the total calculated numbers of immunoglobulin-secreting cells on each array to determine the frequencies of antigen-specific cells. Due to the low frequencies of antigen-specific cells (generally less than 0.1%), it was assumed that any wells containing multiple cells held only 1 cell specific for each antigen. CRM_197_-specific B cells were excluded from analysis and identified as events that stained positive with both CP5-CRM_197_ and CP8-CRM_197_.

### Fluorescence microscopy.

Microengraving data were supplemented with images of the nanowells to determine the occupancy of each well and the viability of each cell. Following microengraving, cells (within the nanowell arrays) were stained with the fluorescent viability dye calcein AM (Invitrogen) at 0.25 µg/ml. Images were collected using an epifluorescence microscope (Zeiss) and software developed in-house (D. Loginov, unpublished data).

### Antigen stimulation and cytokine measurement by Luminex analysis.

For antigen stimulation experiments, macaque PBMCs were thawed, rested overnight, and plated at a concentration of 500,000 cells/well. Cells were stimulated with 8 µg/ml of each antigen in AIM-V media for 48 h. Supernatants were harvested, and the concentrations of the following cytokines were measured: granulocyte-macrophage colony-stimulating factor (GM-CSF), TNF-α, IL-1β, IL-4, IL-6, MIG, vascular endothelial growth factor receptor (VEGF), HGF, EGF, IL-8, IL-17, MIP-1α, IL-12, IL-10, fibroblast growth factor-Basic (FGF-Basic), IFN-γ, granulocyte colony-stimulating factor (G-CSF), monocyte chemoattractant protein 1 (MCP-1), IL-15, IP-10, MIP-1β, eotaxin, RANTES, IL-1RA, I-TAC, MIF, MDC, IL-5, and IL-2. Cytokines were measured using a cytokine Monkey Magnetic 29-plex panel for a Luminex Platform kit (Thermo Fisher) following the manufacturer’s instructions.

### Assessment of antigen-specific antibody responses to vaccination. (i) Opsonophagocytic assay (OPA).

Serological responses to capsular polysaccharides CP5 and CP8 were measured by opsonophagocytic assay (OPA) as previously described (15). Briefly, serial dilutions of heat-inactivated immune sera were mixed with either a CP5- or CP8-expressing clinical isolate of S. aureus and allowed to opsonize the bacteria. The reaction mixtures were then mixed with baby rabbit complement (Pel-Freez) and neutrophil-like HL-60 cells. An OPA antibody titer was defined as the reciprocal of the highest serum dilution resulting in a 50% reduction of the number of bacterial colonies after incubation for 60 min at 37°C compared to the serum-free background control. Samples without detectable activity at the lowest serum dilution of 100 were assigned OPA titer values of 50.

### (ii) Competitive Luminex immunoassay (cLIA).

Competitive immunoassays were used to quantify antigen-specific binding antibodies elicited by the investigational vaccine. The assays monitor the ability of each vaccine component to elicit antibodies that can compete with the binding of antigen-specific monoclonal antibodies (MAbs) that have shown functional activity either *in vitro* or *in vivo*. This approach provides insight into the function and protectiveness of the antigen-specific antibodies. The ClfA MAb prevents binding of live S. aureus to fibrinogen (36), while the MntC MAb inhibits manganese uptake (62). The MAbs used for the CPs facilitated killing of S. aureus as measured by OPA. The cLIA is based on the Luminex MagPlex xMAP technology platform. Spectrally distinct microspheres are coated individually with each of the following antigens, resulting in a mixture of distinct microspheres: CP5, CP8, recombinant ClfA (rmClfA), and recombinant P305A (rP305A). Antigen-coated microspheres are incubated overnight with appropriately diluted serum samples. A mixture of PE-labeled CP5-, CP8-, rmClfA-, and rP305A-specific mouse monoclonal antibodies (MAbs) is then added to the microsphere/serum mixture, and after incubation, the unbound components are washed off. The presence of the fluorescent protein coupled to the monoclonal antibodies allows measurement of the antibody bound to the antigen-coated microspheres by the Bio-Plex reader. Signals expressed as median fluorescence intensities (MFIs) were generated, and the data were converted to units per milliliter using four-parameter logistic (4PL) curves from the reference standard serum, with assigned antibody titers expressed in units per milliliter. This is a competitive assay, and the magnitude of the fluorescent PE signal is inversely proportional to the amount of antigen-specific antibodies in the sample.
