# Supplementary figures and images for: Two Vaccines for Staphylococcus aureus Induce a B-Cell-Mediated Immune Response
Source: mSphere. 2018 Aug 22;3(4):e00217-18. doi: 10.1128/mSphere.00217-18 (PMC6106056; doi:10.1128/mSphere.00217-18)

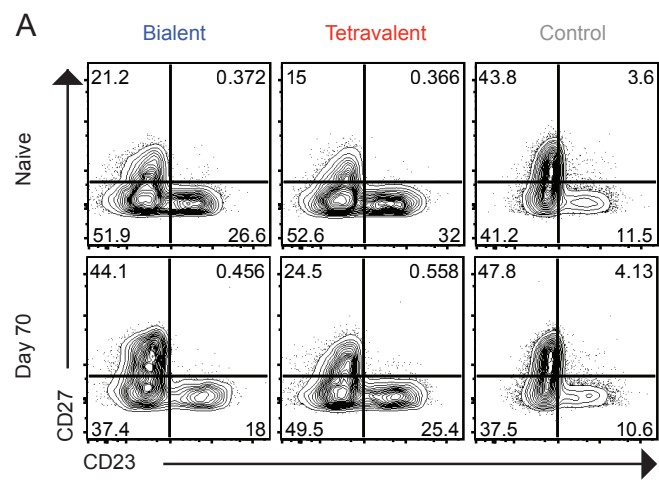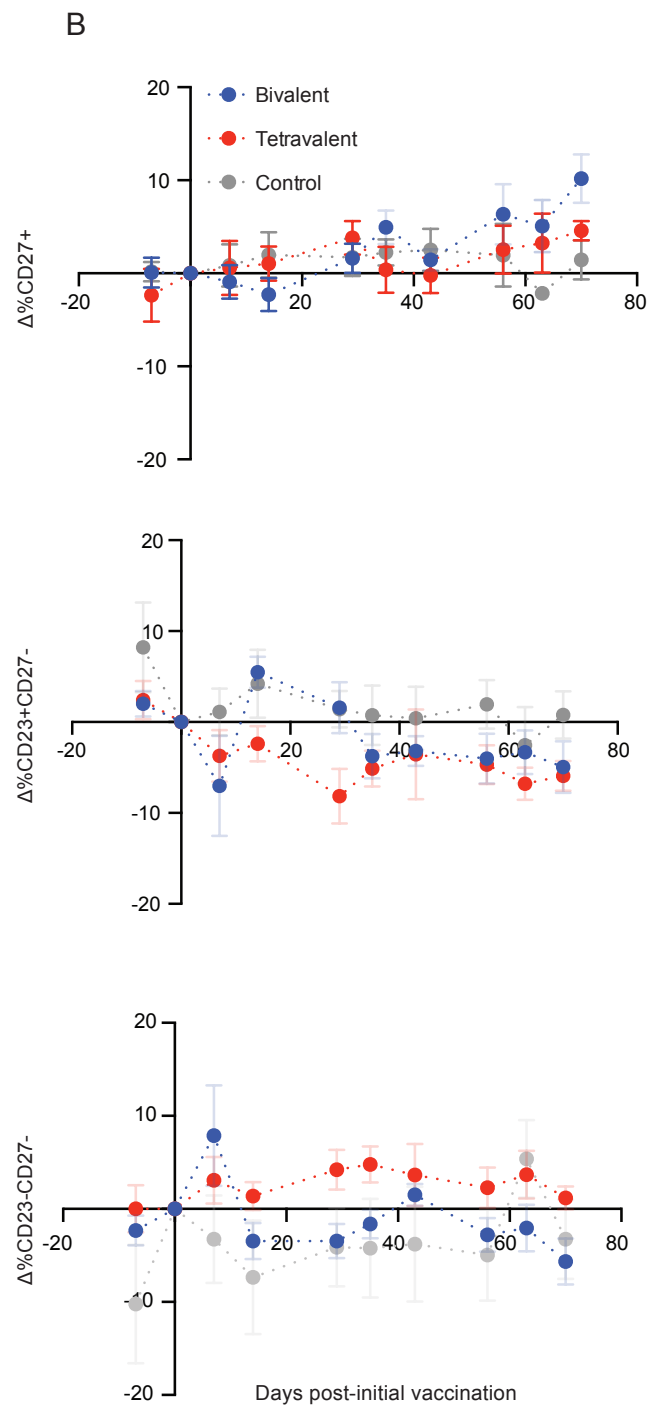

Figure S1

Supplement: FIG S1 [file sph004182620sf1.pdf]

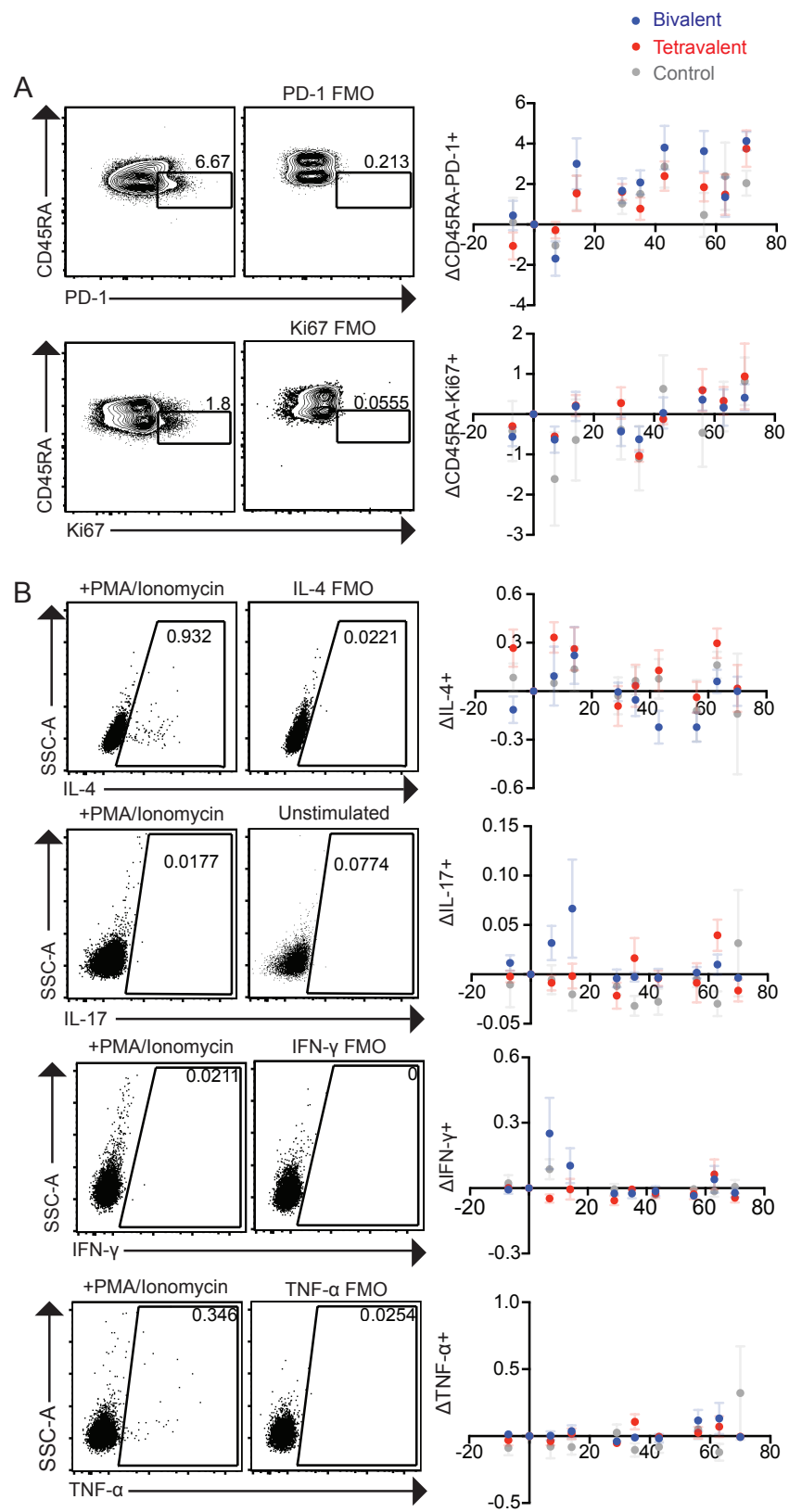

Figure S2

Supplement: FIG S2 [file sph004182620sf2.pdf]

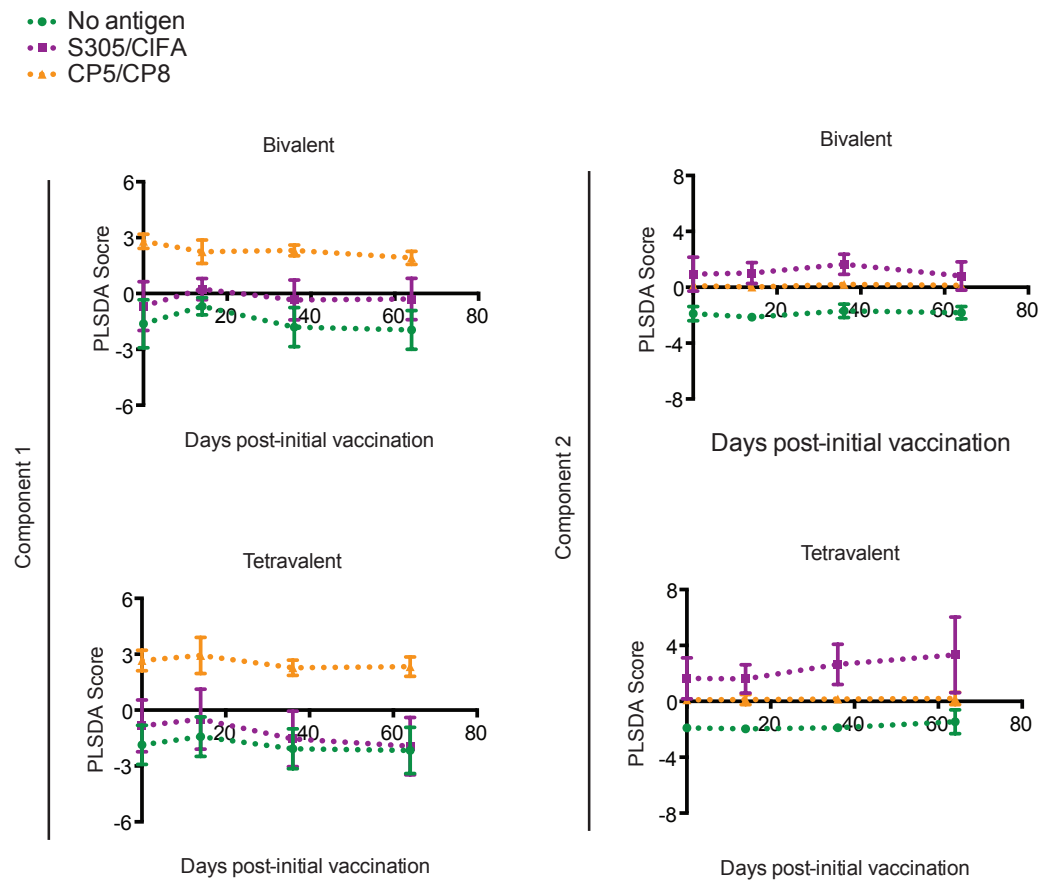

Figure S3

Supplement: FIG S3 [file sph004182620sf3.pdf]

-○- Bivalent  
 -□- Tetravalent  
 -△- Vaccine Buffer  
 Control

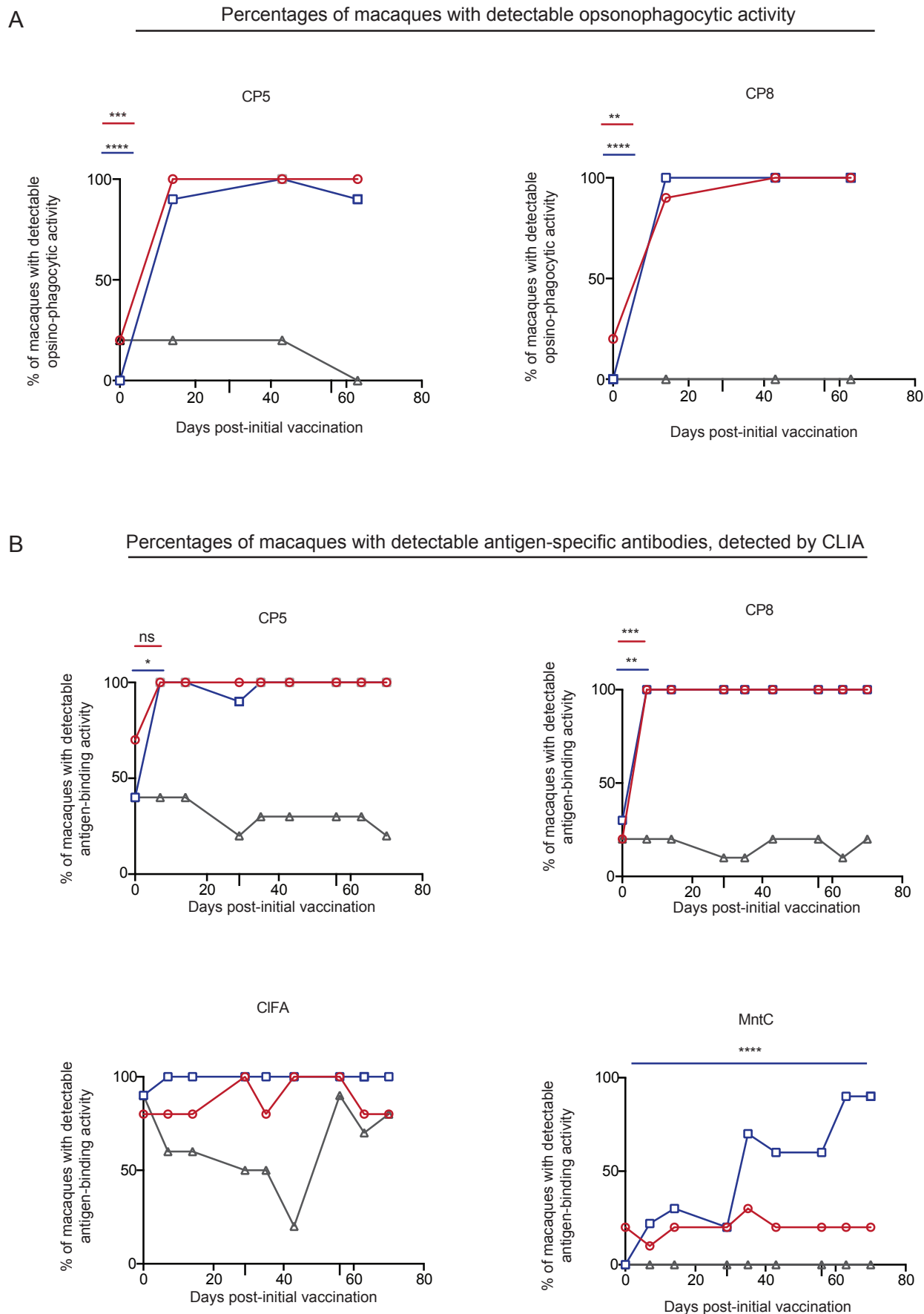

Figure S4

Supplement: FIG S4 [file sph004182620sf4.pdf]
